# Lessons for Robotics From the Control Architecture of the Octopus

**DOI:** 10.3389/frobt.2022.862391

**Published:** 2022-07-18

**Authors:** Dominic M. Sivitilli, Joshua R. Smith, David H. Gire

**Affiliations:** ^1^ Department of Psychology, University of Washington, Seattle, WA, United States; ^2^ Astrobiology Program, University of Washington, Seattle, WA, United States; ^3^ Paul G. Allen School of Computer Science and Engineering, University of Washington, Seattle, WA, United States; ^4^ Department of Electrical and Computer Engineering, University of Washington, Seattle, WA, United States

**Keywords:** soft robotics, octopus, robotic control, biomimetics, neural control architecture

## Abstract

Biological and artificial agents are faced with many of the same computational and mechanical problems, thus strategies evolved in the biological realm can serve as inspiration for robotic development. The octopus in particular represents an attractive model for biologically-inspired robotic design, as has been recognized for the emerging field of soft robotics. Conventional global planning-based approaches to controlling the large number of degrees of freedom in an octopus arm would be computationally intractable. Instead, the octopus appears to exploit a distributed control architecture that enables effective and computationally efficient arm control. Here we will describe the neuroanatomical organization of the octopus peripheral nervous system and discuss how this distributed neural network is specialized for effectively mediating decisions made by the central brain and the continuous actuation of limbs possessing an extremely large number of degrees of freedom. We propose top-down and bottom-up control strategies that we hypothesize the octopus employs in the control of its soft body. We suggest that these strategies can serve as useful elements in the design and development of soft-bodied robotics.

## Introduction

The octopus’s movement is not limited by joints or a rigid skeleton. With the ability to bend its eight arms in any direction at any point along their length, the octopus’s space of possible configurations vastly exceeds that of skeletal animals such as vertebrates or arthropods.

The octopus brain outsources much of the circuitry necessary to control its arms into the arms themselves, where a network of ganglia coordinates sucker and arm behavior with limited feedback from the brain (Young, 1971).

Scientific interest in the octopus has found an application in the development of flexible, adaptable robots (Walker et al., 2005; Calisti et al., 2011; Margheri et al., 2012; Walker, 2013; Nesher et al., 2014; Krieg et al., 2015; Nakajima et al., 2017; Mazzolai et al., 2019). Like the octopus arm, the movement of such robots is hyper-redundant—the high number of degrees of freedom for the robot vastly exceeds those necessary to accomplish most tasks (Walker et al., 2005; Trivedi et al., 2008).

With its ability to combine extreme flexibility with precise manipulation and locomotion, the octopus represents an effective solution for the significant control problems facing soft robotics (Walker et al., 2005; Calisti et al., 2011; Levy et al., 2019). Much of the modern work in octopus motor control has been focused on understanding how the octopus controls its arms despite their vast configuration space, and how this can be applied in the development of soft robotic controllers (Walker et al., 2005; Calisti et al., 2011; Nesher et al., 2014; Mazzolai et al., 2019).

Here we will describe the neuroanatomical organization of the octopus peripheral nervous system and discuss how this distributed neural network is specialized for effectively mediating decisions made by the central brain and the control of limbs possessing an extremely large number of degrees of freedom. We will describe the hierarchical organization of information in this system (Wells, 1978; Zullo et al., 2009), and propose three neuromechanical mechanisms within this organization that reduce the computation necessary for generation of the octopus’s arm behavior: 1) Hierarchical hybrid action selection, which allows for simple motor commands from the brain to be integrated with sensory information from the arms, 2) ascending recruitment, a mechanism of multi-sucker and multi-arm coordination which serves as a strategy for novelty-detection and as an adaptive filter for mechanosensory input, and 3) contact-based navigation and manipulation, which, along with other sensory cues, constrain the degrees of freedom of the octopus’s limbs to a limited range of configurations. We will likewise discuss how these mechanisms can serve as control strategies in soft-robotics.

## Existing Approaches in Robotics

### Robotics Terminology

Typical rigid robots are described in terms of rigid links connected by joints, which allow motion. Rotary joints, for example, allow the angle between links to change, while prismatic joints allow the length of a link to change. If a robot has N joints, then the pose (or configuration) of the robot can be described using a vector of N values, each representing one of the robot’s joint values. The task space or the work space of the robot is the three dimensional space in which it operates. The configuration space is an N-dimensional mathematical space whose axes represent the potential values of the robot’s joint angles. Each pose of the robot can be represented as a single point in this space. A robot’s trajectory through time can be represented as the motion of this point through the configuration space. Kinematics refers to computing the position and orientation of all the robot’s joint angles; inverse kinematics means computing the joint angles necessary to bring the robot’s end effector (for example) to a particular position and orientation (or pose) in the workspace. Obstacles that have a simple geometry in task space, such as a flat table surface or a wall, lead to constraints in configuration space with very complex geometries. The high dimensionality of configuration space, together with the complex geometry of constraints in configuration space, makes robotic planning (the selection of sequences of actions) a difficult computational problem. For an octopus with a very large number of degrees of freedom, the conventional robotics approach to planning and control appears to be computationally intractable. The process of modeling and predicting motion at higher velocities where inertia must be considered is referred to as dynamics in the robotics literature. The computational challenges posed by the octopus are great even using a simplified kinematic framework that ignores dynamics; the computational problems that arise when dynamics is considered become even more challenging.

### How Many Degrees of Freedom Does an Octopus Arm Have?

It is usual to categorize a rigid robot arm by the number of joints or degrees of freedom, or using the terminology above, the dimensionality of its configuration space. Therefore it is natural for roboticists to wonder about the configuration space of an octopus arm: how many dimensions is it? How many degrees of freedom does an octopus arm have? We suggest that the question must be refined. We will propose to discuss the octopus arm in terms of several types of degrees of freedom. The octopus arm is highly compliant: it can be deformed simultaneously at a vast number of independent locations. Thus we might say that it has a very large number of passive degrees of freedom. These are not actuated or directly controllable; rather, when the arm presses against a rigid object, the passive degrees of freedom allow the arm to conform to that object at a large number of points. At another extreme, the octopus exhibits certain arm-scale behaviors that are analogous to robot arms: it bends at a small number of pseudo-joints, which are analogous to rotary joints in a robot arm; and sections of the arm can elongate or shorten, which could be modeled as a small number of prismatic joints. Thus the arm could potentially be characterized by a relatively small number of global arm state degrees of freedom, which would be analogous to the configuration space of a conventional rigid robot arm (One significant difference, however, is that the octopus can change the number of rotary joints dynamically). The octopus arm is also capable of mechanical impedance modulation: adjusting its stiffness above and below a bend. And it can control torsion, rotating around the arm’s axis.

The octopus also has a large number of locally controlled degrees of freedom. Each sucker is controlled by local neural circuitry within the arms. In addition to controlling the sucker pose, this circuitry also innervates and activates the surrounding arm musculature (Gutfreund et al., 2006), which generates the forces and shape of the arm (Kier and Smith, 1985).

We believe that the large number of locally controlled degrees of freedom is a key feature of the octopus that is not present in today’s robots; the primary inspiration for robots we propose to take from the octopus is the use of locally controlled degrees of freedom to simplify planning and control for the arm.

Like the octopus, today’s soft robots have a large number of passive degrees of freedom. Thus this feature of the octopus is known in parts of robotics (specifically, in soft robotics). There are also many rigid robots which use long range visual sensing followed by planning to generate reaching and grasping behaviors in an arm with a small number (often seven or less) of active degrees of freedom. The problem of using long range visual sensing to plan for a small number of active degrees of freedom is computationally tractable; the problem of using vision and planning to control a large number of active degrees of freedom is much harder (apparently intractable) computationally. Controlling a large number of degrees of freedom becomes computationally tractable by making them passive and using contact-based interaction to control them. Thus the octopus’s centrally planned behaviors also are similar to known techniques in robotics. The feature of the octopus that differentiates it most clearly from today’s robots is the use of a large number of locally controlled degrees of freedom. Challenges for robotics include the mechanics and sensing required to build such systems, as well as understanding their function at an algorithmic level.

### Task Domains in Robotics

Robotics can be divided into task domains and capabilities which are necessary to perform these tasks. Grasping typically means using a robot hand or gripper to immobilize an object relative to the robot arm, so that the robot can move the object. Manipulation is a more general term that indicates any robot-induced change to the state of one or more objects. The sequence of operations of grasping, lifting, moving, and setting down are examples of a manipulation procedure. In-hand manipulation means changing the pose or state of an object (for example by rotating it) without setting it down. Manipulation operations often involve multiple objects, such as inserting a peg in a hole or a key in a lock.

The task domain of navigation and locomotion encompasses moving within an environment. For example, a wheeled robot might navigate using the following capabilities: sensing the environment with lidar to determine the locations of obstacles, the perceptual process of building a map of the environment, planning a route on the map that brings the robot to the target location without collision, and executing the plan. Executing the plan involves actuation, the generation of the locomotive force using electric motors, and low level feedback control to overcome errors such as wheel slippage.

The field of soft robotics aims to create robots with mechanical properties and actuation capabilities that are similar to the octopus, in particular compliance. This paper does not focus on actuation per se; this topic is covered thoroughly in reviews on soft robotics (Kim et al., 2013; Galloway et al., 2016; Manti et al., 2016; Polygerinos et al., 2017; Cianchetti et al., 2018; Whitesides, 2018). The paper also does not focus on sensing; since the technological substrates are so different, it is difficult to extract inspiration for today’s engineered systems from octopus sensing. Instead, the paper focuses on the computational level: planning and control, where we believe that system-level inspiration can be most readily extracted today.

### Behavioral Architectures for Robotic Systems

The most widely accepted architecture for robotic systems is known as “sense, plan, act” (Nilsson, 1982). In this paradigm, sensors transduce physical signals, perceptual processes build models of the physical world, planning processes search through the space of potential actions to generate sequences expected to lead to favorable outcomes, and then the best sequence of actions is executed. In the “purest” form of sense-plan-act, the entire plan would be executed “open loop,” in other words without further sensing or control. The main disadvantages of an open loop approach are that the perceptual and planning processes are computationally demanding and brittle or error prone. A closed loop control process is able to compensate for sensing or actuation errors, ideally overcoming perturbations to restore the robot’s state to the planned trajectory.

Proposed alternatives to the sense-plan-act approach include the more biologically inspired subsumption architecture (or reactive or behavior-based robotics) (Brooks, 1986; Payton, 1986; Agre and Chapman, 1987; Firby, 1987; Brooks, 1990). This approach couples sensors more directly to actuators in tight control loops while higher level computational processes modulate and compose these lower level behaviors. Advantages of this approach include fast and dynamic robot behavior, the ability to respond to dynamically changing environments, lower computational requirements, and insensitivity to modeling errors, since the robot does not construct an explicit model of the environment. In the purest form of this approach, the environment functions as its own representation; rather than the robot considering its own internal computational model of the environment, the robot would consult its own sensors to collect required information about the state of the world at the present moment. Disadvantages of this approach are that the behaviors tend to be “greedy,” and thus less intelligent than approaches that are able to avoid local minima. Greedy is a term used in computer science to describe an algorithm in which, at each time step, the action is selected that provides the greatest reward in that time step. Explicit planning approaches are able to delay gratification, selecting actions in the present with lower immediate rewards, but with higher rewards predicted later. For example, consider a simple robotic scenario in which a robot, which knows its position, is attempting to navigate to a goal by following a map. Faced with a branch in the road, the greedy approach is to always select the road that (at the particular location where the choice must be made) heads most closely in the direction of the goal. However, without the capability to look ahead on the map, this can lead to the robot becoming stuck in a dead end. A smarter, non-greedy approach would consider the future benefits of choosing a particular sequence of road choices. This can allow the robot to avoid getting stuck in dead ends.

Low level fast reactive control has been explored for integration with robot manipulators in proximity perception (Mayton et al., 20102010; Navarro et al., 2021) as well as visual servoing (Espiau et al., 1992).

Recently Model Predictive Control (MPC) has seen a resurgence in robotics. MPC is a version of the sense-plan-act approach but modified to be more reactive and dynamic. In MPC, the sensing and planning steps occur as usual, but only the first step of the action plan is executed. Then the sense-plan-act cycle starts again. The frequent sensing and re-planning allow the robot to react dynamically to changes in the environment, while still making more intelligent choices than a purely reactive system (Erez et al., 2011; Lenz et al., 2015; Zhang et al., 2016; Liu et al., 2017; Williams et al., 2017; Gillespie et al., 2018; Amos et al., 2019; Kabzan et al., 2019). In terms of computational resources, it requires even more than the conventional sense-plan-act cycle, since plans are being constantly generated and re-generated. We will discuss how the control architecture of the octopus may provide benefits reminiscent of MPC but with lower computational costs.

### Underactuated Robotics

Underactuated robotics (Spong et al., 1998; Reyhanoglu et al., 1999; Birglen et al., 2008) makes use of systems in which the number of individually controllable degrees of freedom is less than the number of degrees of freedom of the mechanism. For example, robot hands have been designed with just one actuated degree of freedom (one motor), but multiple joints (Odhner et al., 2014; Deimel and Brock, 2016). When the robot contacts an object to be grasped, it conforms to the object’s shape. We will discuss ways that the octopus uses an analogous strategy.

### Contact-Rich Dynamics

It is typical in robotics to sense and plan in order for the robot to avoid collisions with obstacles or objects. There are some exceptions which explicitly consider planning and control through contact. In manipulation, contact is necessary, but is avoided as long as possible, and the planning process tends to focus on the motions before contact. Legged locomotion is another area in which contact (between the leg and the ground) must be considered, and most of the robotics work that considers contact originates from legged locomotion (Erez et al., 2011; Posa et al., 2014; Marcucci et al., 2017; Pace and Burden, 2017; Deits et al., 2019).

### Planning on Constraint Manifolds

Even simple motion constraints, such as a planar table top or wall, produce complex geometries in the configuration space of a robot arm with several degrees of freedom. A robot arm moving its end effector along a table top corresponds to motion within a lower dimensional sub-space of the arm’s full configuration space; this subspace is known as a constraint manifold. The general problem of generating motion plans that remain within such a constraint manifold is a challenging computational problem because of the high dimensionality of the space and the complexity of the geometry (Berenson et al., 2009; Berenson et al., 2011). For an octopus to use a centralized approach to generate a motion plan that moves its arm along a complex surface would likely be computationally infeasible: the number of degrees of freedom of the octopus arm is much higher than a typical articulated robot arm and many parts of the octopus arm touch the surface simultaneously. In the next section we describe the approach that appears to be used by the octopus, and discuss the potential for robots to make use of this strategy.

### Hybrid Control

Hybrid control refers to hierarchically organized systems that choose among discrete control modes at higher levels, and for each of these modes, different continuous controllers operate at lower levels (Branicky et al., 1998; Goebel et al., 2009). The term hybrid refers to the combination of discrete and continuous dynamics. The control strategy of the octopus shares some features with hybrid control.

## Mechanical Properties of the Octopus

The octopus’s arms, like elephant trunks, earthworm bodies, and vertebrate tongues, are muscular hydrostats (Kier and Smith, 1985; Kier and Stella, 2007). Unlike skeletal muscle, which relies on skeletal elements to support the generation of movement, muscular hydrostats generate force and serve as the support for movement. In the octopus arm these muscles are arranged in transverse, longitudinal and oblique (helical) orientations. The requirement of the hydrostat to maintain constant volume ensures that when the transverse muscles decrease the cross-sectional area of the arm, the arm increases in length. Likewise, as the longitudinal muscles contract and the arm shortens, the cross-sectional area increases. The interplay of the three muscle groups and the lack of inherently rigid structure equips the arm with its remarkable flexibility (Kier and Smith, 1985; Kier and Stella, 2007; Kennedy et al., 2020).

The capacity for robots to generate adaptive behavior can be facilitated by exploiting their material properties. Elasticity, for instance, can stabilize the body during motion, while compliant properties can allow an effector to adapt to the shape of an object for grasping and manipulation (Pfeifer et al., 2014). These characteristics can be both energetically and computationally favorable. As muscular hydrostats capable of complex motion over a vast range of configurations, the octopus arm serves as a particularly interesting model for the study and implementation of material properties in control architectures.

Within the arrangement of the arm’s muscles are embedded arrays of collagenous connective tissue fibers (Kier and Stella, 2007; Fossati et al., 2011). The material properties of the collagen fibers give this tissue inherent stiffness, and to some degree extensibility and elasticity (Gutnick et al., 2011). In muscular hydrostats, these properties can transmit stress during motion, store elastic energy, and provide structural reinforcement (Di Clemente et al., 2021). Di Clemente et al. (2021) investigated the contribution of these properties to the arm’s elasticity and stiffness and the possible roles they play in the arm’s motion. The distinct formation of the fibers within the longitudinal and transverse muscles were shown to give the two muscle groups different mechanical properties. Di Clemente et al. suggest that these properties indicate a possible role of longitudinal muscles in energy storage and shock absorption, and transverse muscles in maintaining posture and resisting deformation. Overall such passive properties can locally modulate behavioral responses while minimizing their energetic cost and alleviating the computational need for neural feedback (Di Clemente et al., 2021). Not only can mechanical properties of the arm facilitate computation, some evidence points to the potential of these properties to serve a direct computational role (Nakajima et al., 2013; Nakajima et al., 2014; Nakajima et al., 2015). This potential role of both biological and artificial material properties presents fascinating directions for the field of robotics.

## Architecture of the Octopus’s Control System

Most of the octopus nervous system exists within its eight arms (Young, 1971). Down the center of each arm, a nerve cord (known as the arm or axial nerve cord) runs parallel to the suckers, 200–300 of which are staggered down the ventral side of the arm (Young, 1965; Gutfreund et al., 2006). The nerve cord consists of a dense, continuous network of neural circuitry (neuropil) which projects from a surrounding layer of unipolar nerve cell bodies. The neuropil enlarges at the base of each sucker. These enlargements are commonly referred to as ganglia and the sections between them as the interganglionic regions. As with other elements of the octopus’s peripheral anatomy, there has been some inconsistency in the terms used for these elements. Such terms include arm ganglia (Zullo et al., 2011), axial ganglia (Rowell, 1963; Rowell, 1966), brachial ganglia (Graziadei, 1965b; Graziadei and Young, 1971; Gutfreund et al., 2006), sucker ganglia (Young, 1963; Young, 1965; Altman, 1968), and medullary cord (Zullo et al., 2019). We will refer to these elements as brachial ganglia. These ganglia serve as local sensorimotor integration centers for their corresponding suckers and nearby arm musculature, and account for about 350 million of the octopus’s over 500 million neurons (Young, 1963; Rowell, 1966; Budelmann and Young, 1985). Two bundles of nerve fibers called the axonal tracts run along the nerve cord dorsal to the ganglia. At the base of the arm the axonal tracts fuse and continue to the brain as the brachial nerve, which serves as a pathway through which the ganglia communicate with the brain via the axonal tracts. The brachial ganglia are connected to their immediately distal and proximal neighbors through the neuropil (Graziadei and Young, 1971), and there is some evidence suggesting a connection through the axonal tracts (Altman, 1968). The eight brachial nerves of the arms converge on the brachial lobe of the brain. Where the brachial nerve and the nerve cord meet, the interbrachial commissure interconnects the nerve cords of the arms into a neural ring, allowing communication between arm networks independent of the brain (Altman, 1968).

**FIGURE 1:**
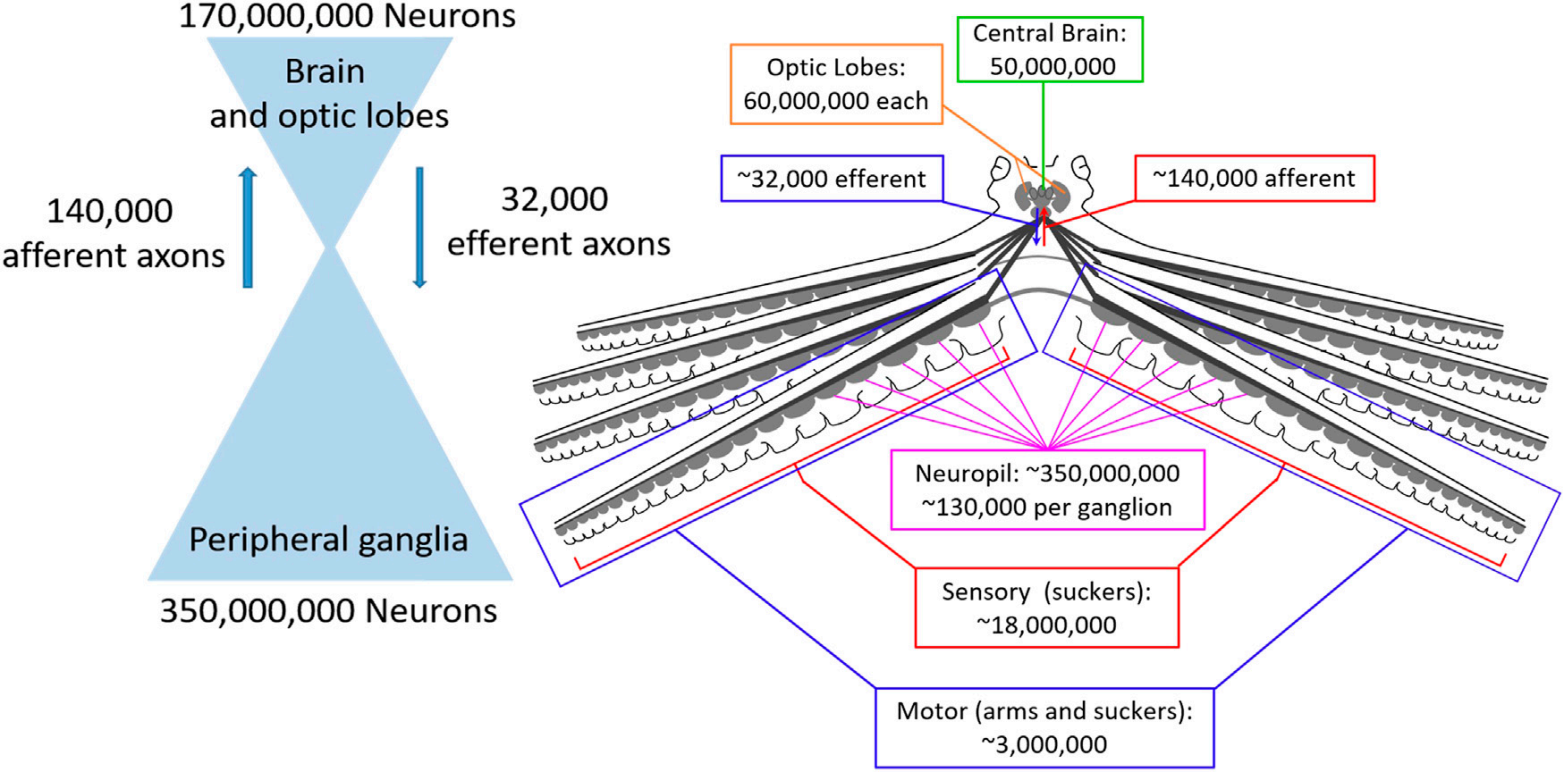
Numbers of neurons and axonal connections in the octopus nervous system. Left: The octopus nervous system is composed of two large populations of neurons, the central brain (optic lobes and brain, 170 million neurons) and peripheral ganglia (350 million neurons) that are connected by orders of magnitude fewer neurons (140 thousand afferent and 32 thousand efferent), creating a bottleneck that requires enormous compression of sensory and motor signals. Right: Numbers for each component of the nervous system on an anatomical diagram.

Figure 1 shows the neural architecture of the octopus with estimated neuron and axon numbers indicated. Consistent with ganglia being sensorimotor integration centers, the vast majority of their neurons (120,000 of 130,000) seem to integrate sensory and motor information (Young, 1963; Young, 1965; Rowell, 1966). Following sensory integration in the ganglia there is a dramatic reduction in the number of sensory pathways between the suckers and the brain, with an estimated reduction from 18 million sensory neurons within the suckers to 140 thousand neurons entering the brain from the brachial nerves (Young, 1965).

Similarly, the pathways through which the brain communicates motor commands to the arms are orders of magnitude smaller than the local innervation of the musculature by the ganglia. Collectively, the brachial nerves contain an estimated 32 thousand efferent (outbound) axons, while an estimated 3 million motor neurons terminate on the musculature of the arms and suckers from the neuropil of the nerve cords (Young, 1965; Matzner et al., 2000; Rokni and Hochner, 2002; Nesher et al., 2019).

### Sensing and Perception

The octopus’s distributed nervous system supports a complex chemotactile system within its arms and suckers. Each sucker contains a dense array of mechanical and chemical receptors, estimated at a density of several tens of thousands of receptors on a sucker 3 mm in diameter (Graziadei, 1965a). By comparison, the human fingertip has a few hundred mechanical receptors in a similar area (Johansson and Vallbo, 1979).

Chemoreceptors are the most abundant of these receptor types, outnumbering the other cell types by a factor of ten (Graziadei, 1965a). These cells aggregate along the rim of the sucker disk, where they are appropriately placed for contact with dissolved substances in the surrounding water. Chemical information is transmitted to higher neural centers after ascending through multiple levels of integration (Graziadei and Gagne, 1976).

While the outer rim of the sucker is specialized for the transduction of chemical information, the sucker disk is specialized for transduction of mechanical information, including texture, shape, and perhaps the integrity of adhesion (Graziadei and Gagne, 1976).

Information acquired by the suckers have been shown to follow two primary pathways (Rowell, 1963; Rowell, 1966; Altman, 1968; Gutfreund et al., 2006; Zullo et al., 2011). These pathways run through the neuropil between the ganglia and through the axonal tracts.

The pathway through the neuropil seems to carry information proximally and distally, and transmit information from the sucker sensory fields and proprioceptive information from local musculature between brachial ganglia (Young, 1963). This pathway is evidently polysynaptic, such that information along this pathway is subject integration with other sources of sensory and motor information. This pathway seems to be able to relay signals over long distances of the arm provided intermediate ganglia aid in propagating the signal (Altman, 1968).

The neuropil pathway likely supports the ability of suckers to recruit their neighbors. This recruitment behavior, retained in isolated arm preparations (Rowell, 1963; Altman, 1968; Gutfreund et al., 2006; Zullo et al., 2011), enables sensory input to a given sucker to result in neighboring suckers and musculature bending toward the activated sucker, an effect which can propagate along the arm if these neighboring suckers are likewise activated. This mechanism has several advantages that we will discuss throughout the text.

The second pathway along the axonal tract seems to also transmit information proximally (Zullo et al., 2011) and distally (Rowell, 1966). The proximal signal appears to carry information to the brain while the distal-traveling signal is believed to recruit the distal arm toward proximal stimuli (Rowell, 1966). Rowell (1966) reports a considerable range of spatial representation and sensitivity among the afferent units in this pathway (Rowell, 1966). Some units responded to input to single suckers, while others responded to input to all suckers or specific groups of suckers.

The extreme sensory compression that occurs from ganglia to brain suggests that the ganglia may actively filter sensory input to determine which signals reach the brain. Since stimulus relevance depends on context, such as ambient fluid turbulence or substrate irregularity, the peripheral network may contain a mechanism of normalization by which sensory input is weighted proportionately to the global level of input for the entire animal. A rock encountered on a flat surface, for instance, would be more likely to induce a behavioral response than a rock encountered on a rocky surface. A possible mechanism to accomplish this could be a mutually inhibitory signal sent between suckers that is proportionate in strength to the level of stimulation of the suckers. This could serve as a useful mechanism to work in parallel to sucker recruitment to determine and prioritize the most relevant information encountered by the suckers.

### Proprioceptive Information Is Locally Integrated in Ganglia

Octopuses possess multipolar cells that resemble muscle receptors seen in other species (Graziadei, 1964). While proprioceptive information about local movement and muscle position is found within the ganglia, this information, including the relative position of the suckers, has not been demonstrated within the afferent pathways in the axonal tracts or the brain (Wells and Wells, 1957; Wells, 1964; Rowell, 1966). Wells (1964) suggested that mechanical transduction occurs through the degree of distortion of the suckers upon a surface and regularity of the surface’s texture. It is possibly due to this mechanism that the octopus is not able to distinguish orientations of textures and the octopus’s ability to distinguish simple three-dimensional shapes is inhibited by cutting grooves into their surface (Wells, 1964). The axonal tract evidently does not communicate autonomous movement by the suckers or the arm (Rowell, 1966). Wells also found evidence that octopuses failed to learn to discriminate the weight of objects handled by the arms (Wells, 1961), suggesting that this information is also absent from the higher neural centers.

Despite these findings, Gutnick et al. (2020) showed a form of proprioceptive learning based on octopus’s increased preference in reaching for the rewarded side of a confined two-choice arm maze, suggesting that the brain has some representation of and control over the directionality of the arm during extension (i.e. the arm’s horizontal and vertical angle or yaw and pitch) without visual information. Octopuses can also learn to open jars containing a food reward more efficiently over multiple trials (Fiorito et al., 1990) and adapt their feeding technique with clams through trial-and-error (Anderson and Mather, 2007), suggesting the retention of some proprioceptive information from this kind of task.

Proprioceptive information is evidently exchanged between arms through the interbrachial commissure (Graziadei, 1965b; Altman, 1968), revealing a potentially important role of this pathway in the arms’ ability to coordinate during movement. This is especially interesting given that this information is largely absent from the brain.

## Action Selection

In “sense, plan, act” models motor signals would originate centrally and precisely control movement. However, rather than generating motor output as specific patterns of muscle activation, the central brain of the octopus seems to broadly transmit general behavioral programs (Zullo et al., 2009). While behaviors are seemingly decided in the brain, the motor circuitry for executing these behaviors exist within the arm nerve cords. The details of where and how to execute these behaviors may then be locally determined by integrating mechanical, chemical, and proprioceptive information within the brachial ganglia (Zullo et al., 2019). Consistent with this model, motor pathways within the arms are largely autonomous. Reaching, sucker adhesion, probing, recoiling from aversive stimuli, and sucker recruitment can all be readily evoked in arms separated from the brain (Rowell, 1963; Graziadei, 1965b; Rowell, 1966; Altman, 1968; Sumbre et al., 2001; Gutfreund et al., 2006; Zullo et al., 2011; Hague et al., 2013; Katz et al., 2021) and movement in these isolated arms kinematically resembles that seen in whole animals (Sumbre et al., 2001).

This peripheral organization of motor circuitry bears some resemblance to the spinal cord in vertebrates. Here neural circuits responsible for rhythmic movement such as locomotion, and reflexes which, for example, lead to avoiding tissue damage and maintaining posture, can operate with little to no intervention from the brain (Lemon, 2008). On the other hand, an adaptation unique to primates of a monosynaptic pathway from the motor cortex to densely organized motor neurons allows for a unique capacity for dexterity among their hands and fingers (Pearson and Gordon, 2013). This example is seemingly antithetical to the organization of the octopus, for which efferent pathways from the brain appear to innervate large pools of motor neurons along the length of the nerve cord (Zullo et al., 2019). The differences in degrees of freedom, stereotypy, feedforward versus feedback activation, generation of rhythmic movement, the role of top-down and bottom-up modulation, and the levels of polysynaptic integration present interesting points of comparison between these two control systems that we hope will be explored in depth in the future.

**FIGURE 2:**
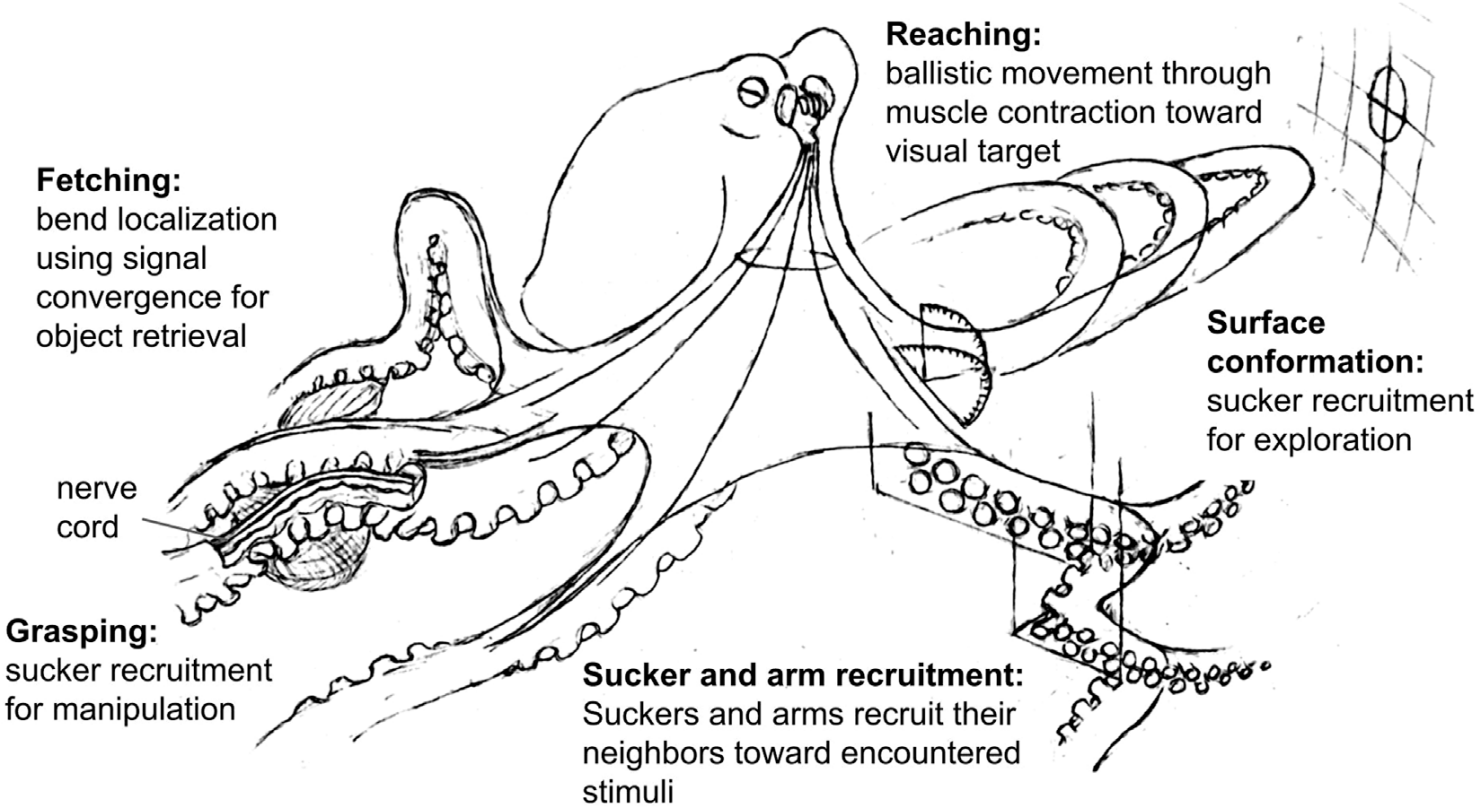
Octopus arm control strategies. Fetching: from the base of the arm, an outbound wave of muscle activation converges with another inbound wave determined by the location of the object (Sumbre et al., 2006). Arm musculature is activated at this midpoint, bending the arm appropriately to pass the object proximally. Sucker recruitment: in response to a stimulus, suckers recruit their neighbors to bend toward this stimulus. These suckers can then recruit their neighbors as this mechanism continues down the arm (Rowell, 1963; Altman, 1968; Gutfreund et al., 2006; Zullo et al., 2011). Arm recruitment: in response to stimulation of one arm, the corresponding suckers on neighboring arms orient toward the site of stimulation (Graziadei, 1965b; Altman, 1968). Grasping: as suckers collectively adhere to an object, sucker recruitment provides multiple afferent pathways for sensory input and multiple efferent pathways for manipulation. If the suckers find prey during foraging, the suckers will recruit their neighbors to capture and immobilize the animal (Rowell, 1963; Altman, 1968; Gutfreund et al., 2006; Zullo et al., 2011). Surface conformation: as suckers recruit their neighbors toward encountered surface features, the arm’s shape conforms to that of the surface (Altman, 1968; Kennedy et al., 2020). Reaching: using visual information the brain determines the horizontal and vertical angle (yaw and pitch) of the arm. The arm then extends by a wave of muscle contraction resembling a propagating bend toward the visual target (Gutfreund et al., 1998; Sumbre et al., 2001; Richter et al., 2015).

During the generation of movement, the octopus brain appears to send signals to the nerve cords that activate the motor circuits for different behaviors (e.g. reach, fetch, reject), and these behaviors may then be modified based on peripheral sensory information. For example, bend propagation (see Figure 2) has been shown to begin mid-way down the arm if the arm is reaching through a narrow opening (Richter et al., 2015), and the bend location during fetching behavior is seemingly determined by where the object of interest is along the arm (Sumbre et al., 2006). The amount of information that these behavioral signals carry with them appears to vary between behaviors. The reaching signal, for example, seems to include yaw and pitch of the arm (Gutfreund et al., 1998; Sumbre et al., 2001; Gutnick et al., 2020) while behaviors that are retained in isolated arms, such as sucker recruitment, may rely primarily on sensory feedback from the suckers and require less information from the brain. Recent work has shown efferent pathways of the axonal tract making broad, non-specific contacts with ganglia along the arm, supporting the idea that the brain does not precisely control specific segments of the arms (Zullo et al., 2019). The control of sucker movement and adhesion is also a local operation of the peripheral ganglia, though is subject to broad, top-down regulatory signals from the brain that are not directed to specific suckers (Altman, 1968). Together, this evidence suggests that the brain is limited in its ability to precisely control the arms, and relies on behaviors that are coordinated locally within the arms with minimal feedback from the brain. Figure 2 summarizes the examples of these behaviors that have been described. We predict that the brain may initiate and recall combinations of behavioral signals that lead to more complex sequences of behavior, such as manipulation (Fiorito et al., 1990; Anderson and Mather, 2007).

Fine-scale local control through sucker recruitment (section below) enables precise grasping and manipulation of objects when they are encountered by the suckers. As suckers are recruited to collectively adhere to an object, the control the arm has over the object is compounded. Through locally recruiting suckers to interact with an object, this mechanism adaptively scales the number of afferent pathways for sensory information and efferent pathways for manipulation, all without precise central control.

## Locomotion

Despite the limited bandwidth through which the brain and arms communicate, the arms together demonstrate remarkable coordination during locomotion (Levy et al., 2015). This movement is independent of the orientation of the body: the octopus can change direction of crawling without changing orientation of its body and vice versa. Although arms were found to individually generate rhythmic patterns of movement, the pattern between arms showed no obvious consistency. The octopus appears to lack the rhythmic motor output that characterizes central pattern generator-driven locomotion of other animals. The recruitment of the pushing movement in the arms to generate locomotion may result from a moment-to-moment pattern of activation from the brain, allowing the octopus to immediately adjust its direction (Levy et al., 2015).

Coordinated behavior between arms has been shown to be retained following isolation from the brain (Graziadei, 1965b; Altman, 1968), and severing an arm’s connections to the interbrachial commissure has shown to affect the arm’s ability to coordinate with the other arms during locomotion (Altman, 1968). These observations suggest that the peripheral nervous system is to some degree responsible for coordinating arm behavior. The interbrachial commissure, which connects the nerve cords into a ring, is the primary pathway by which information from the arms bypasses the brain (Altman, 1968) and information carried by the interbrachial commissure evidently includes a representation of the spatial arrangement of the arms (Graziadei, 1965b; Altman, 1968). These findings suggestion that this pathway may play an important role in the ability of the arms to work in cooperation during complex behaviors, including the pattern of arm recruitment during locomotion.

## Octopus-Inspired Robotics

While, like any natural system, there is a considerable nuance to the octopus’s control system, we would like to propose three broad control strategies that the octopus appears to be employing, and which are applicable to the current field of robotics: hierarchical hybrid action selection, used as a top-down control strategy, ascending recruitment, used as a bottom-up control strategy, and contact-based navigation and manipulation which emerges from recruitment.

### Hierarchical Hybrid Action Selection

**FIGURE 3:**
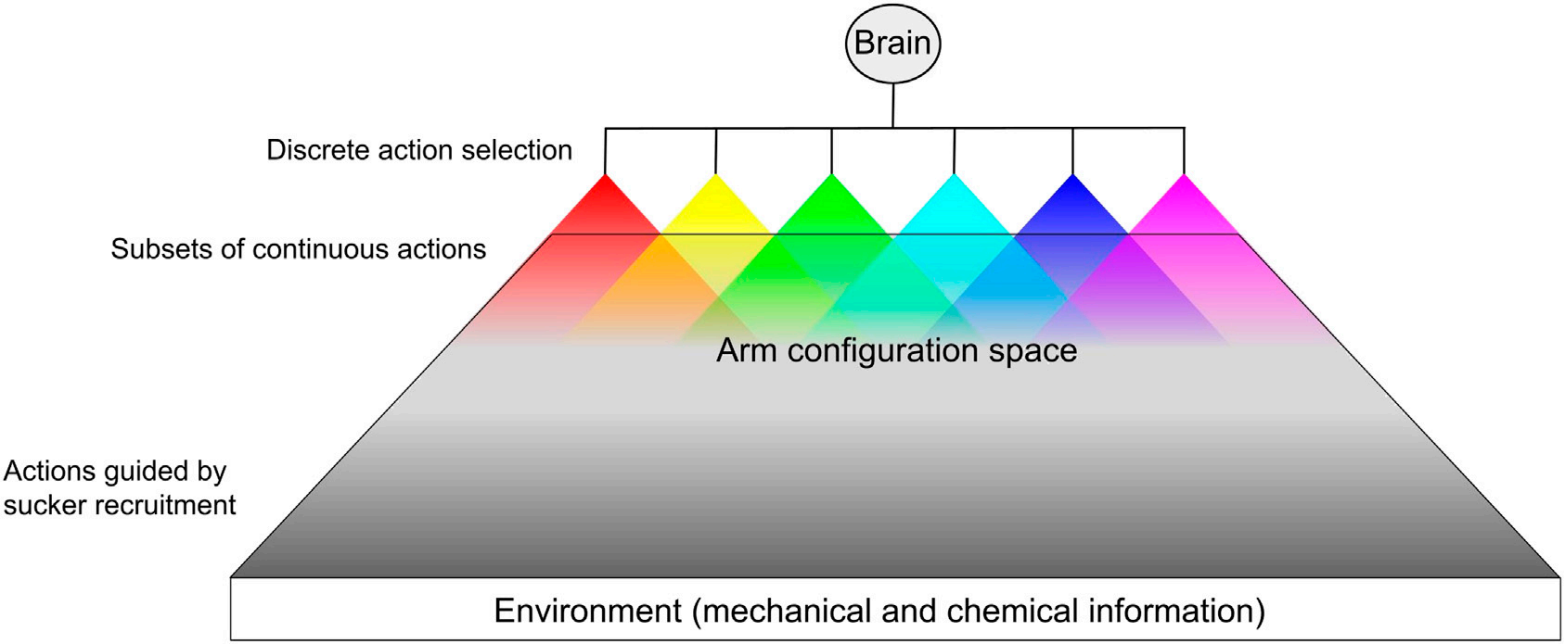
Hypothetical pipeline of hybrid hierarchical action selection. For each arm, the brain determines an action over a discrete domain (e.g. fetch, reach, push, reject, etc.). For each discrete action, the arm is allowed a subset of continuous stereotyped actions executed based on peripheral proprioceptive information and sensory information from the environment. The actuation of these continuous action subsets overlap within the arm’s configuration space. Most of the arm’s configuration space is dominated by the possible arm shapes resulting from sensory-guided sucker recruitment (e.g. surface conformation). Some behaviors, such as reach, have some continuous parameters that the brain may be able to set (Gutnick et al., 2020).

The octopus controls a soft body with a large number of passive degrees of freedom, complex musculature, and highly concentrated sensory fields. We propose that this control problem is simplified by organizing motor commands into a hierarchical structure of action selection (Zullo et al., 2009; Merel et al., 2019), thereby reducing the state space to manageable levels. As the high-level controller, the brain of the octopus selects from among actions over a discrete domain. The brain then sends a general signal to the peripheral network of ganglia, which is composed of subordinate semi-independent agents (the ganglia). These agents then select from a subset of actions (local motor control) over a continuous domain as defined by the action decided by the brain, similar to hybrid control strategies (Branicky et al., 1998). Figure 3 illustrates this form of control in the octopus. The control problem inherent in the octopus’s large number of degrees of freedom could be further simplified by relying on feedforward movement strategies in unconstrained (high-dimensional) conditions (e.g. reaching toward a visual target (Sumbre et al., 2001)), and feedback strategies when operating in a lower-dimensional constrained environment (Gutnick et al., 2011), in which the arm can use the mechanical and chemical composition of its surroundings as a reference [e.g. searching through crevices (Mather and O’Dor, 1991; Forsythe and Hanlon, 1997)]. It is tempting to identify exploration as the default motor strategy of the arms based on observations of denervated arms readily engaging in probing and sucker recruitment. These mechanisms may be overridden in favor of locomotory behaviors.

Hierarchical control has been suggested as a general framework for the control of complex systems (Barto and Mahadevan, 2003), and the octopus is an exemplar of this form of control (Zullo et al., 2009; Wayne and Abbott, 2014). While hierarchical control theory is an attractive framework for understanding motor control across numerous species (Merel et al., 2019), a striking feature of the octopus is that the putative levels of hierarchical control are physically separated and connected by distinct axonal pathways.

### Ascending Recruitment and Contact-Based Control

Sucker recruitment serves as an effective mechanism in foraging, exploration, and manipulation. We propose that by relying on this simple sensory-motor reflex across such a breadth of behaviors, the octopus can simplify the control of its highly flexible arms.

Suckers that encounter a stimulus recruit unoriented suckers, and in conflicting situations where two or more stimuli are recruiting a shared set of suckers, presumably the stronger stimulus will send the stronger signal and override the competing stimuli. Although unknown, a mechanism for such “winner-take-all” sucker recruitment could be realized by a mutually inhibitory signal sent between suckers proportionate to the strength of the stimulus that they have encountered.

**FIGURE 4:**
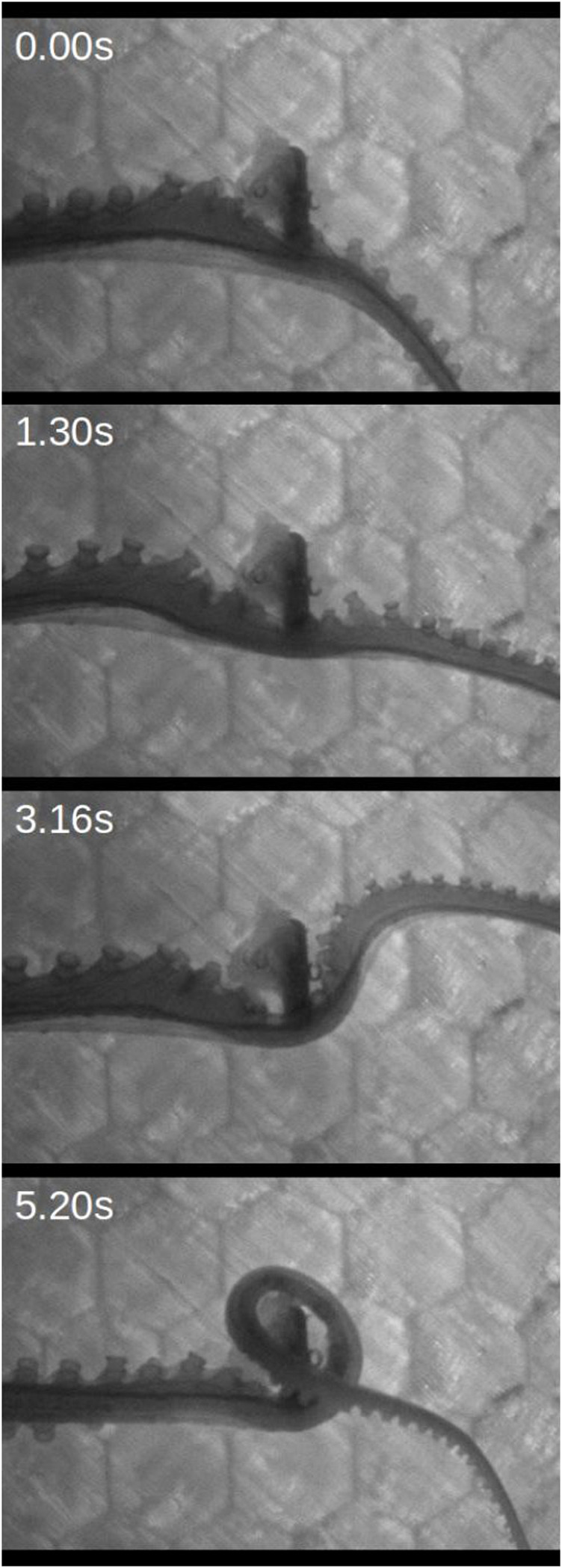
Sucker recruitment used to grasp shrimp meat during foraging. Consistent with localized spread of sucker recruitment, orientation and movement of suckers towards the food occurs in waves propagating from the point of food contact. See also Supplementary Video S1.

The suckers may therefore act as an adaptive sensory filter by locally prioritizing stimuli, providing a peripheral mechanism for determining which signals are sent across the narrow bandwidth through which the brain and the arms communicate. While the representation of a stimulus encountered by a single sucker is limited in higher neural centers, a stimulus that successfully attracts the attention of multiple suckers by eliciting a recruitment signal would be represented through multiple afferent pathways. Engaging multiple suckers with a stimulus both maximizes the amount of information acquired from the stimulus and the amount of control the suckers have over it. Spreading sucker recruitment could also determine which of the suckers on a given arm act as the end effectors during manipulation and locomotion. This mechanism also works as an effective hunting strategy: if a sucker finds prey, it will recruit its neighbors to efficiently capture and immobilize the animal. This may be particularly effective if the less prominent distal suckers find prey and recruit larger proximal suckers to aid in capture. This mechanism can additionally benefit foraging by conforming the arm to surface features of the environment. In this case, recruitment could lead the arm around corners or into crevices to find prey (Mather and O’Dor, 1991; Forsythe and Hanlon, 1997), enabling an exhaustive search over even the most complex surfaces. See Figure 4 and Supplementary Video S1 for an example of sucker recruitment during food detection and retrieval.

Traditional robotics expends a great deal of computation to avoid collisions with obstacles. The vast configuration space of the octopus arm appears to compound this problem, providing a nearly infinite number of configurations for which a collision with environmental features must be considered. However, behavioral and neural evidence suggests that the octopus avoids this seemingly intractable computational problem; instead, the octopus’s reliance on contact as a result of sucker recruitment could simplify its control strategy, mechanically restricting its configuration space to the contraint manifolds defined by obstacles, dramatically reducing the computational complexity of its control scheme.

Two additional varieties of sensory input have been shown to possibly restrict the arms’ degrees of freedom. Nesher et al. (2014) discovered that a chemical in the octopus’s skin prevents the suckers from attaching to it. This mechanism is evidently peripheral—it is retained in the arms when severed, but can seemingly be overridden by the brain when the arms are left intact. Katz et al. (2021) found that arm tips reflexively withdraw from light, a response which is likely mediated by the brain but acts independently of visual feedback. Both chemical and photosensory feedback therefore seem to play a similar role as mechanical input in limiting the arms’ range of possible configurations.

Graziadei (1965b) and Altman (1968) noted that stimulation of one arm will cause the nearest arm to turn toward the site of stimulation, even without innervation from the brain. This represents a possible second level of recruitment (Byrne et al., 2006) that could result in the same benefits of sucker recruitment. Recruitment of neighboring arms, like that of the suckers, could rally multiple effectors for handling objects and immobilizing prey, and compound the strength of the afferent signal communicated to the brain. The brain could then update the motor plan across multiple arms based on this locally-filtered and amplified signal. The pathway through the brain provides an additional means by which suckers may recruit each other. In this case, the brain may be considered another recruitable winner-take-all node in this ascending recruitment mechanism that influences behavior across the entire network by generating a renewed motor plan. At this level, additional factors such as visual information, memory, and internal state also contribute to updating the motor plan.

Afferent pathways within the arms carrying mechanical information are fast adapting and habituate quickly to unchanging stimuli (Rowell, 1966). Dynamic mechanical stimuli are thus preferentially communicated between ganglia and along the axonal tract to the brain. Novelty, possibly representing fluid motion, surface irregularity, or prey movement, could therefore serve as a strong ascending recruitment signal across the hierarchy of the octopus nervous system. This may represent a neural mechanism for the notable curiosity the octopus displays (Mather and Anderson, 1999; Kuba et al., 2003; Kuba et al., 2006a; Kuba et al., 2006b), and the motivational connection between exploration and foraging (Kuba et al., 2006a). The octopus’s nervous system supports seeking out and assessing the novelty of information in the environment, which is an area of active research in robotics (Grizou et al., 2020). We suggest it is appropriate, therefore, that particular attention is given to the octopus in the design of robotics for the purposes of exploration, with application to projects such as the Honda Curious Minded Machine program.

## Conclusion

### Lessons From the Octopus for Robotics

An underactuated robot hand can bring multiple joints into a configuration that closely mirrors the object’s geometry, despite not having enough control degrees of freedom to generate this pose in the absence of the object. Octopuses appear to make even more extensive use of under-actuation and compliance: they are underactuated at both the mechanical level and the control level. Mechanically, the body of the octopus is highly compliant, enabling it to conform to complex geometries with computationally simpler control than would be required for active control of non-compliant mechanisms.

Unlike most rigid robots, which are usually programmed to avoid collisions with obstacles, the octopus arm appears to seek out contact with surfaces or other nearby objects. We hypothesize that contact with external rigid objects allows the arm to localize itself with respect to the environment. This is similar to a strategy called coastal navigation that has been employed in mobile robotics to reduce positional uncertainty (Roy et al., 1999). Due to the arm’s many passive degrees of freedom (i.e. its mechanical compliance), the same set of motor commands resulting in sucker recruitment can cause the arm to conform to a large number of different surface geometries. Thus one simple motor program could potentially produce arm shapes that mirror a wide variety of surfaces.

The reliance on contact with the environment, its local distributed control of suckers, and a highly compliant body, enables the octopus to accomplish complex behaviors using much less computation than would be required by a brute force sense-plan-act approach to planning and control for its large number of actuated degrees of freedom. The octopus generates motor commands via a hierarchical process, with higher level motor commands originating in the brain and lower level closed loop control processes occurring at the suckers. At the lowest, mechanical level, the octopus makes use of underactuation via its highly compliant body; this is analogous to some work in underactuated hands, and soft robotics. The local control of suckers, including recruitment, appears to be an intermediate strategy between passive mechanical compliance and global computational planning that may be the most novel compared to conventional robotics. While it is common for robots to use a slower planning process to choose position, velocity, or torque commands for joints, and faster control loops to implement those commands while rejecting disturbances, the octopus has a more complex layer of distributed, local control, which allows it to control thousands of actuated degrees of freedom, namely the sucker and arm musculature. The octopus’s highly capable distributed control layer appears to be quite distinct compared to conventional robotics. Even though it is not possible today to build a robot with as many locally controlled degrees of freedom as an octopus, the architecture of the octopus could be implemented today in a robot with a smaller number of locally controlled degrees of freedom. Achieving a better understanding of the functional capabilities provided by the octopus’s hierarchical control scheme, as well as understanding the limitations of its local distributed layer, suggests new approaches to planning and control of robots, approaches which have the potential to provide more capability with less computation.
